# Childhood Pneumonia Screener: a concept

**DOI:** 10.15172/pneu.2015.6/646

**Published:** 2015-12-01

**Authors:** Jukka Rasanen

**Affiliations:** Department of Anesthesiology, Lee Moffitt Cancer Center, Tampa, Fl USA

**To the Editor:**

We thank Dr Grimwood for his interest and thoughtful comments on our concept of a ‘smart’ phone based Childhood Pneumonia Screener [1]. The comments are directed in particular to the third phase of the evaluation, an outcome study. Much development and deployment work needs to be conducted before we get to this phase, and the specifics of the outcome study have not yet been formulated. Therefore, Dr Grimwood’s comments are quite timely and helpful.

The suggestion of a cluster-randomised design is interesting and relevant. We do not yet have a good understanding of the complexity of deployment for this technology and how easy it might be to reach out to a sufficient number of communities. Porco et al [2] used 48 communities randomised into four arms; our outcome study could probably be conducted with fewer given the two arms, screener and control. Dr Grimwood is correct in his suggestion of including outcome measures other than mortality. We anticipate that by the time the system is functional, we will have a good understanding of what can and should be additionally measured.

Partial deployment of this technology beyond the peripheral healthcare facility to the parents and caregivers would indeed be a clever addition to the project. It would, as Dr Grimwood points out, address another significant problem in childhood pneumonia case flow: the failure of caregivers to recognize the severity of the child’s illness. The feasibility of a ‘smart’ phone application naturally depends on the availability of phones and the required infrastructure in the community, but these already are surprisingly widely distributed and over the time span of this project will spread even more.

**Competing interests:** The author declares no competing interests.
